# Fate of Chloromethanes in the Atmospheric Environment: Implications for Human Health, Ozone Formation and Depletion, and Global Warming Impacts

**DOI:** 10.3390/toxics5040023

**Published:** 2017-09-21

**Authors:** Wen-Tien Tsai

**Affiliations:** Graduate Institute of Bioresources, National Pingtung University of Science and Technology, Pingtung 912, Taiwan; wttsai@mail.npust.edu.tw; Tel.: +886-8-770-3202; Fax: +886-8-774-0134

**Keywords:** chloromethanes, toxicity, environmental property, atmospheric degradation, environmental exposure risk

## Abstract

Among the halogenated hydrocarbons, chloromethanes (i.e., methyl chloride, CH_3_Cl; methylene chloride, CH_2_Cl_2_; chloroform, CHCl_3_; and carbon tetrachloride, CCl_4_) play a vital role due to their extensive uses as solvents and chemical intermediates. This article aims to review their main chemical/physical properties and commercial/industrial uses, as well as the environment and health hazards posed by them and their toxic decomposition products. The environmental properties (including atmospheric lifetime, radiative efficiency, ozone depletion potential, global warming potential, photochemical ozone creation potential, and surface mixing ratio) of these chlorinated methanes are also reviewed. In addition, this paper further discusses their atmospheric fates and human health implications because they are apt to reside in the lower atmosphere when released into the environment. According to the atmospheric degradation mechanism, their toxic degradation products in the troposphere include hydrogen chloride (HCl), carbon monoxide (CO), chlorine (Cl_2_), formyl chloride (HCOCl), carbonyl chloride (COCl_2_), and hydrogen peroxide (H_2_O_2_). Among them, COCl_2_ (also called phosgene) is a powerful irritating gas, which is easily hydrolyzed or thermally decomposed to form hydrogen chloride.

## 1. Introduction

In the 20th century, halogenated aliphatic hydrocarbon, including chlorofluorocarbons (CFCs), chloromethanes (e.g., carbon tetrachloride), chloroethanes (e.g., methyl chloroform, and hydrochlorofluorocarbons (HCFCs), were extensively used for commercial and industrial uses, like refrigerants, cleaning solvents, and fire extinguishing agents. However, these volatile organic compounds (VOCs) can be transported to the stratosphere where they are readily photolyzed by ultraviolet (UV) radiation to release chlorine atoms. The catalytic chain reaction of chlorine atoms will cause depletion of stratospheric ozone. Under the terms of the Montreal Protocol on Substances that Deplete the Ozone Layer first established in 1987 [1], the production of these so-called ozone-depleting substances (ODS) has been phased out by several time schedules. In addition to causing the destruction of stratospheric ozone, the release of halogenated VOCs will contribute to global warming, meaning that these so-called greenhouse gases (GHGs) possess significant potential for absorbing the infrared (IR) radiation reflected from the surface of the Earth [2,3,4]. On the other hand, the presence of hydrogen (H) in some halogenated VOCs may trigger photochemical oxidation reactions by highly-reactive radicals (e.g., hydroxyl radicals) in the lower atmosphere, which can generate toxic degradation products, such as ozone (O_3_) and hydrogen peroxide (H_2_O_2_) [5].

Chloromethanes, including methyl chloride (CH_3_Cl), methylene chloride (CH_2_Cl_2_), chloroform (CHCl_3_), and carbon tetrachloride (CCl_4_), are currently used in a broad range of applications because of their excellent physicochemical properties [6]. Among them, methylene chloride may be the most commonly used solvent in metal finishing and vapor degreasing processes. Chloroform is primarily used as a solvent in the pharmaceutical industry, and also as a monomer for the production of fluoropolymers, like polytetrafluoroethylene (PTEF). In view of this, environmental and health effects of chloromethanes have been extensively studied [7,8,9,10,11]. The presence of carbon-chlorine bonds and the electron withdrawal by chlorine atoms will affect the chemical properties of chloromethanes and their toxicological profiles. Such concerns have arisen as a result of their toxicity and their environmental fates in the atmosphere. To minimize the potential impact on human health, all chloromethanes have been listed as hazardous air pollutants (HAPs) under the U.S. Clean Air Act Amendments [12]. More noticeably, the assessment studies have led to the inclusion of some chloromethanes for their carcinogenicity potential by international organizations, such as the International Agency for Research on Cancer (IARC), the National Toxicology Program (NTP) of the U.S. Department of Health and Human Services, and the American Conference of Governmental Industrial Hygienists (ACGIH).

Except for carbon tetrachloride (CCl_4_), chloromethanes are controlled neither by the Montreal Protocol nor the Kyoto Protocol. Thus, these short-lived VOCs are present in the atmosphere, and their tropospheric abundances have increased significantly in recent years [13,14]. This article is a brief overview on chloromethanes regarding their chemical and physical properties, industrial and commercial uses, and impacts to human health. In addition, the environmental properties of chloromethanes and their atmospheric degradation mechanisms in the literature are also summarized and further discussed in this paper.

## 2. Chloromethanes

### 2.1. Chemical and Physical Properties

Methane is an inert compound, representing the simplest alkane due to its tetrahedral (*sp^3^*) structure with four equivalent carbon-hydrogen (C-H) bonds. However, progressive chlorination of methane will yield chloromethanes (or chlorinated methanes), including chloromethane (methyl chloride), dichloromethane (methylene chloride), trichloromethane (chloroform), and tetrachloromethane (carbon tetrachloride). The presence of the carbon-chlorine (C-Cl) bond will affect their chemical/physical properties and toxicological profiles because the electron-withdrawing capacity of chlorine atom significantly increases the electrophilicity of the carbon atom [7]. As a consequence, the relatively low binding energy of the C-Cl bond in chloromethanes results in relatively high chemical reactivity. In the study on thermal degradation of chloromethane mixtures [15], CH_3_Cl was found to be the most thermally stable compound, and CHCl_3_ was to be the most thermally labile compound under oxidative and pyrolytic conditions. As listed in Table 1 [12,16,17,18,19,20,21,22,23], density, boiling point (b.p.), viscosity, refractive index, and partition coefficient all increase with increasing chlorine content, while vapor pressure, flammability, dipole (or dielectric constant), water solubility, and latent heat of vaporization at the b.p. indicate a decreasing trend with increased chlorine content.

### 2.2. Industrial and Commercial Uses

Owing to their specific and solvent properties, chloromethanes are widely used in industrial and commercial applications, ranging from metal cleaning and vapor degreasing operations to reaction media for chemical synthesis processes. Moreover, they are used as chemical intermediates for a variety of products [18]. It should be noted that CH_3_Cl was believed to come mainly from natural sources, including biomass burning, tropical plants, and biological processes in the oceans [9]. However, the pressure to reduce emissions of VOC (especially carbon tetrachloride) to the environment has led to a decrease in demand for their applications in recent years. In 2000, the data on the consumption are given below: 1249 × 10^3^ tons for CH_3_Cl, 328 × 10^3^ tons for CH_2_Cl_2_, 554 × 10^3^ tons for CHCl_3_, and 20 × 10^3^ tons for CCl_4_ [18]. In comparison with the data in 1990 (i.e., 869 × 10^3^ tons for CH_3_Cl, 392 × 10^3^ tons for CH_2_Cl_2_, 450 × 10^3^ tons for CHCl_3_, and 536 × 10^3^ tons for CCl_4_), the consumption of methyl chloride has significantly increased because it is mainly used as a raw material for the manufacture of silicone polymers.

The primary use of methyl chloride is in the silicone manufacturing sector. Less common uses include the feedstock for the production of methyl cellulose, quaternary amines, butyl rubber, and agricultural products. Methylene chloride is a volatile liquid, which is used both as a solvent and a chemical intermediate. As a chlorinated solvent, it is extensively used as a degreasing and paint/adhesive remover (or stripper) in the metal finishing industry. It is also used as a reaction media and extraction solvent for the production of a variety of pharmaceuticals (drugs) and food products. Furthermore, it has been applied to the production of hydrofluorocarbons, and used as a low-temperature heat-transfer medium in air-conditioning systems. Prior to the mid-1990s, chloroform was primarily consumed in the manufacture of chlorofluorocarbons (e.g., CFC-22) and hydrochlorofluorocarbons (e.g., HCFC-22), which were used as refrigerants. As the Montreal Protocol entered into force on 1 January 1989, its current consumption patterns focus on a solvent in the pharmaceutical industry, and an intermediate for producing fluoropolymers, such as polytetrafluoroethylene (PTFE). Carbon tetrachloride is mainly used as a fire extinguisher and as an intermediate in the production of chlorofluorocarbons (e.g., CFC-11 and CFC-12) during the 1980s [6]. However, its use started to decrease in the early 1990s due to the phase-out dictated by the Montreal Protocol. Nowadays, its current uses are still as a special solvent for chemical reactions due to its non-polar nature, and as a chemical intermediate for the production of vinyl chloride from ethylene dichloride (EDC).

### 2.3. Impacts On Human Health

The toxicological profiles and health impacts of chloromethanes have been extensively studied because some chlorinated solvents have been classified as probable or possible carcinogens [7,8,9,10,11]. It is well known that the impacts of chlorinated compounds on human health are estimated in view of their toxicological data, physicochemical properties and exposure risks. Subsequently, the exposure risk of a chemical is dependent on its exposure profiles (e.g., route, concentration, time, and individual) and environmental fate (or environmental behavior). As listed in Table 1, chloromethane exists as a gas in the environment under ambient conditions, but the other three chloromethanes (i.e., methyl chloride, chloroform and carbon tetrachloride) are used in the liquid form as volatile solvents or intermediates. Therefore, these chloromethanes are usually released to the atmosphere from emission sources, including waste streams, contaminated water, and sediments [24]. In this regard, inhalation should be the primary route of human exposure [25]. Skin (dermal) contact and absorption may be other exposure routes in the workplace environment [26].

Table 2 summarizes the health hazards [26] and threshold limit value (TLV) basis and critical effects of chloromethanes [27]. In brief, the most toxic effects of exposure to chloromethanes in human are observed in the central nervous system (CNS), liver, and kidneys. More noticeably, metabolism of methyl chloride to carbon monoxide (CO) can result in the formation of carboxyhemoglobin (CO-Hb), the cause of hemoglobinemia. To protect the health of workers from potential acute and chronic hazards via the inhalation route, the occupational exposure limits (OELs) of these chlorinated compounds have been established by the official agencies (e.g., National Institute for Occupational Safety and Health (NIOSH)) and non-profit professional organizations (e.g., American Conference of Governmental Industrial Hygienists (ACGIH), Deutsche Forschungsgemeinschaft (DFG)). Table 3 lists the OELs of chloromethanes in terms of the time-weighted average (TWA), which were compiled from ACGIH (USA), the Occupational Safety and Health Administration (OSHA, Washington, DC, USA), NIOSH (Washington, DC, USA), DFG (Bonn, Germany), and the Ministry of Labor (OSHA, Taipei, Taiwan). As can be seen, carbon tetrachloride has relatively small OEL-TWA values, suggesting that it should be more toxic to human.

Animal tests on all chloromethanes show positive results for carcinogenesis [7]. In this regard, these chlorinated compounds have been listed as hazardous air pollutants (HAPs) or air toxics by the United States Environmental Protection Agency (US EPA) under the Clean Air Act Amendments (CAAA) [12]. Furthermore, tissue-specific cancers may develop in humans when individuals are chronically exposed to chloroalkanes, especially methylene chloride. Such chronic exposures are likely to occur by inhalation in industrial occupational settings [28]. Cancer sites include lung, nasal cavity, paranasal sinus, liver, and bile duct, as well as leukemia in white blood cells. By compiling data from various agencies worldwide, Table 4 lists the carcinogen classifications relevant to chloromethanes, which are those developed by the International Agency for Research on Cancer (IARC), US EPA, US National Toxicology Program (NTP), ACGIH, and DFG. According to animal experiments, epidemiological studies, and other relevant data, the IARC groups are outlined below.

- Group 1: Carcinogenic to humans;
- Group 2A: Probably carcinogenic to humans;
- Group 2B: Possibly carcinogenic to humans;
- Group 3: Not classifiable to be carcinogenic to humans; and
- Group 4: Probably not carcinogenic to humans.

Other respected carcinogen classifications include ACGIH and DFG, which are intended to provide a practical tool for industrial hygienists in controlling the exposure to airborne pollutants in the workplace environment. The former classifies chemicals into five categories.

- Group A1: Confirmed human carcinogen;
- Group A2: Suspected human carcinogen;
- Group A3: Confirmed animal carcinogen with unknown relevance to humans;
- Group A4: Not classifiable as a human carcinogen; and
- Group A5: Not suspected as a human carcinogen.

Similarly, the latter establishes the following carcinogen classifications.

- Category 1: Substances that cause cancer in humans;
- Category 2: Substances that are considered to be carcinogenic for humans;
- Category 3: Substances that cause concern that they could be carcinogenic for humans, but cannot be assessed conclusively owing to lack of data;
- Category 4: Substances with carcinogenic potential for which genotoxicity plays no or at most a minor role. No significant contribution to human cancer risk is expected provided the MAK value is observed; and
- Category 5: Substances with carcinogenic and genotoxic potential, the potency of which is considered to be so low that, provided a MAK value is observed, no significant contribution to human cancer risk is to be expected

According to the carcinogen classifications in Table 4, they are quite different for chloromethanes. For example, methylene chloride, the most commonly used one, has been classified as “2A” by IARC, but listed in Category 5 by DFG.

## 3. Atmospheric Fate of Chloromethanes

### 3.1. Environmental Properties in the Atmosphere

As shown in Table 1, chloromethanes are gases or very volatile liquids as solvents, and have low relative solubilities in water. Moreover, their octanol/water partition coefficients (Log *P*ow) are lower than 3.0, indicating that they have low potential bioaccumulation in the fatty tissues of living organisms [29]. In view of their physicochemical properties, these chlorinated compounds will tend to partition predominantly to the atmosphere upon release to the environment. As in the case of halogenated VOCs, the main factors determining their atmospheric concentrations are sources and sinks. The sources of these compounds are related to anthropogenic or biogenic processes and subsequent emissions into the atmosphere. By contrast, their sinks are correlated with the atmospheric lifetime, which is defined by its rate of removal in the troposphere or stratosphere. The above implies that the atmospheric lifetime not only determines the global average concentration (or surface mixing ratio) of the organic compound in the atmosphere (seen in Table 5), but also plays an important role in the ozone variations of the troposphere and stratosphere, and the impacts on regional air quality and climate change. Table 5 summarizes the common environmental properties of the four chloromethanes, including CH_3_Cl, CH_2_Cl_2_, CHCl_3_, and CCl_4_ [13,30,31,32,33,34]. These chlorinated compounds contain C-H and C-Cl bands with characteristic infrared (IR) absorption patterns in the atmosphere, suggesting that they are greenhouse gases, tropospheric ozone precursors, and stratospheric ozone-depleting initiators.

#### 3.1.1. Atmospheric Lifetime

When considering the atmospheric fate of a compound, it is useful to obtain their atmospheric lifetimes, which can be calculated from the ratio of the total atmospheric burden to the total atmospheric loss rate due to all processes [35,36], including photochemical reactions in the troposphere and photolysis in the stratosphere. This value can also be estimated by its mixing ratio (atmospheric concentration) and global budget (source strength). In general, VOCs are mainly removed from the atmosphere by photochemical reaction mechanisms, including reactions with hydroxyl radicals (HO·). When taking a typical daytime HO· concentration of approximately 1.0 ×10^6^ radicals/cm^3^ and the rate coefficients for reactions of HO· with these chloromethanes at room temperature (i.e., 298 K) [35,36], their atmospheric lifetimes can be estimated according to a pseudo-first-order loss rate. From the data in Table 5, carbon tetrachloride has a longer atmospheric time (i.e., 33 years) than the other chloromethanes (i.e., 0.4–1.0 years), alkanes and alkenes, but shorter atmospheric lifetimes than the chlorofluorocarbons (45–1700 years) and perfluorocarbons (1000–50,000 years) [33]. These comparative results reflect the electrophilic reaction of HO· to C-H. For example, methane and ethane have atmospheric lifetime of 12.4 years and 1.4 days, respectively [37]. It should be noted that the actual lifetime could be shortened if there are other competing loss processes such as photolysis. On the other hand, the actual concentrations of HO· are variable, depending on geographical locations and time horizons. Thus, one should carefully examine the concentration of HO· when the atmospheric lifetime of an organic compound is cited with respect to HO· attack.

#### 3.1.2. Photochemical Ozone Creation Potential

In the troposphere, ozone (O_3_) is formed under sunlight radiation in the presence of nitrogen oxides (NOx) and VOCs. The formation mechanism of ozone is initiated by the reactions of HO· with VOC molecules. Subsequently, the photochemical reaction is catalyzed by NO_x_. More significantly, tropospheric ozone (or ground-level ozone) is recognized as one of the most important environmental threats to the regional air quality because it is hazardous to human health and can also cause damage to vegetation and a variety of materials. In this regard, shorter-lived VOCs, including alkanes, alkenes, and oxygenated VOCs, will become the main precursors of ozone formation in the urban and regional atmosphere. In order to compare the relative ozone formation potentials of organic compounds, the photochemical ozone creation potentials (POCPs) for many VOCs have been reported in the literature [5,31,38,39,40]. POCP is generally presented as a relative value where the amount of ozone produced from a certain VOC is divided by the amount of ozone produced from an equally large emission of ethene (C_2_H_4_). Generally, the POCP value of an organic compound is compared with that of ethane, whose POPC is defined as 100. According to the definition, calculated POCP values are relative and scenario-based values. A simplified procedure has been developed for estimating POCP from molecular properties of a target compound (i.e., molecular weight, number of carbon atoms, number of C-C and C-H bonds, and the rate coefficient for the reaction with OH radicals at 1 atm and 298 K). POCP values of chloromethanes are listed in Table 5 [31]. Obviously, chloromethanes have relatively low POCP values which lie between 0, for CFCs, and 12.3, for ethane. The presence of hydrogen in chloromethanes may contribute to photochemical ozone formation, but they do not have significant impacts on tropospheric ozone formation, thus exempting them from air quality regulations for unsaturated VOCs and oxygenated VOCs. As described above, released chloromethanes are liable to exist in the atmosphere due to their volatility, low water solubility and heavier vapor density. In a manner similar to those for typical VOCs, methyl chloride (CH_3_Cl), and methylene chloride (CH_2_Cl_2_) are liable to react with HO· to convert them into carbonyl species, like carbon dioxide (CO_2_), CO, and phosgene (COCl_2_). These carbonyl products are believed to be primarily taken up into clouds, followed by hydrolysis [41,42,43]. Hydrolysis forms HF and acids (e.g., formic acid), which are absorbed into the oceans, clouds, and rainwater, decreasing their pH.

#### 3.1.3. Global Warming Potential

It is well known that all VOCs emitted into the atmosphere may cause the Earth’s average temperature to rise. This phenomenon is called the greenhouse effect, which is derived from long-wave radiation absorption, contributing to radiative forcing of climate change. By definition, the radiative efficiency of a molecule means its ability to trap solar heat in the atmosphere, giving a unit of W m^−2^ ppb^−1^. As seen from Table 5, radiative efficiencies of chloromethanes are significantly smaller than those of CFCs, hydrofluorocarbons (HFCs) and fully-fluorinated compounds [33]. In addition to their molecular properties, radiative efficiencies of chloromethanes are relatively low because of their non-uniform horizontal and vertical mixing in the atmosphere [13]. Another approach for calculating radiative forcing due to GHGs is to use GWP. GWP expresses the time-integrated radiative forcing over a given time horizon due to the pulsed (instantaneous) emission of a kg of gas relative to the integrated radiative forcing for the emission of a kg of reference gas (i.e., CO_2_) [35]. Therefore, GWP shows the relative increase in earthward infrared (IR) radiation flux due to the emission of GHGs. As described above, chloromethanes (except for CCl_4_) possess hydrogen atoms, thus leading to an increase in chain reactivity and reduction in their atmospheric lifetimes. As a consequence, methyl chloride (CH_3_Cl), methylene chloride (CH_2_Cl_2_), and chloroform (CHCl_3_) have relatively smaller values of GWP (Table 5) compared with those of CFCs and HFCs. It is noted that GWP value of carbon tetrachloride (CCl_4_) is substantially greater than CO_2_ as seen in Table 5. Hence, CCl_4_ has been listed as an ODS as a result of the Montreal Protocol [1]. Its forced phase-out began on 1 January 2010.

#### 3.1.4. Ozone Depletion Potential

The stratospheric ozone layer plays a vital role in shielding harmful ultraviolet (UV) radiation emitting to the surface of the Earth. In this regard, chlorine atom participates in catalytic ozone destruction cycles in the stratosphere. The Montreal Protocol, signed in 1987, is the first international treaty for protecting the ozone layer by the phase-out of ODS, including CFCs and some halogenated compounds (i.e., halons, CCl_4_, CH_3_CCl_3_, HCFCs, CH_3_Br). As a result of the successful implementation of the Montreal Protocol, the levels of stratospheric chlorine are declining [13,14]. Although chloromethanes (except for CCl_4_) still contain hydrogen, the release of the chlorine atom to the stratosphere is expected to be small compared with CFCs, because they are readily attacked by HO· in the lower atmosphere (troposphere), limiting their transport through the troposphere to the stratosphere. As seen from Table 5, these solvents or chlorinated VOCs have short atmospheric lifetimes and relatively small ozone depletion potentials (ODP) [5,33]. Herein, ODP of a compound is defined as the ratio of the global loss of ozone from that compound to the loss of ozone by a reference compound (i.e., CFC-11) at a steady state per unit mass emitted. Thus, ODP provides a relative measure of the potential for each organic compound to affect stratospheric ozone. 

### 3.2. Atmospheric Degradation Mechanism

Chlorinated aliphatic hydrocarbons are now receiving much attention because of their toxicological profiles, environmental properties, and atmospheric concentrations. For example, using atmospheric model simulations [14], the contribution of methylene chloride, not controlled under the Montreal Protocol, to stratospheric ozone depletion merits attention owing to its marked increase in recent years. It has been recognized that removal processes of fully-halogenated compounds, such as CFCs and CCl_4_, will occur in the stratosphere where the powerful UV light photolyzes them, thus, releasing chlorine atoms and subsequently involving a cycle of ozone destruction. By contrast, the fate and transport of common hydrocarbons in the troposphere are well known because the presence of C-H and other susceptible bonds may contribute to the formation of oxidants and other degradation products by photochemical reactions with some highly-reactive species.

As described below, the atmospheric degradation reactions of chloromethanes (except for CCl_4_) and other ozone-depleting substances are initiated by hydroxyl radical (HO·) attack, giving an alkyl radical (R·) which will promptly add oxygen to produce an alkylperoxy radical (RO_2_·) [30,44]:

RH + HO· → R· + H_2_O (RH=CH_3_Cl, CH_2_Cl_2_, or CHCl_3_)

R· + O_2_ → RO_2_·

Alkylperoxy radicals (RO_2_·) in the lower atmosphere react primarily with nitrogen monoxide (NO) and hydroperoxyl radical (HO_2_·) [35]. The former mainly produces alkoxy radical (RO·) and nitrogen dioxide (NO_2_):

RO_2_· + NO → RO· + NO_2_

Moreover, alkylperoxy radicals can react with NO_2_ to form a peroxynitrate (RO_2_NO):

RO_2_· + NO_2_ → RO_2_NO_2_

The abovementioned reaction is reversible in that peroxynitrate thermally decomposes or photolyzes in the reverse of this reaction:

RO_2_NO_2_ → RO_2_· + NO_2_

RO_2_NO_2_ + hν → RO_2_· + NO_2_

Another fate of alkylperoxy radical is to react with a hydroperoxyl radical, forming a hydroperoxide (RO_2_H):

RO_2_· + HO_2_· → RO_2_H + O_2_ (or RO· + HO· + O_2_)

Hydroperoxide can photochemically decompose to alkylperoxy radicals, or thermally photolyze to alkoxy radical and hydroxyl radical.

RO_2_H + HO· → RO_2_· + H_2_O

RO_2_H + hν → RO· + HO·

However, hydroperoxide will be generally degraded to carbonyl compounds, such as COCl_2_, CO, and CO_2_. As for the atmospheric fates for methyl chloride (CH_3_Cl), methylene chloride (CH_2_Cl_2_), and chloroform (CHCl_3_), their major halogenated products include inorganic chlorine, formyl chloride (HCOCl), and phosgene (COCl_2_), which are further described below [41,44]:

1. Methyl chloride (R=CH_2_Cl)
  (1) CH_2_ClO· → HCOH + Cl
  (2) 2CH_2_ClO_2_· → 2CH_2_ClO· + O_2_
    CH_2_ClO· + O_2_ → HCOCl + HO_2_·
    CH_2_ClO· + HO_2_· →HCOCl + H_2_O_2_
  (3) HCOCl → CO + HCl
    HCOCl + Cl → COCl + HCl
    COCl + O_2_ → CO_2_ + ClO
    COCl → CO + Cl
    2ClO + O_2_ →O_2_ + 2Cl
2. Methylene chloride (R=CHCl_2_)
  (1) CH_2_ClO· → HCOCl + Cl
  (2) 2CHCl_2_O_2_· → 2CHCl_2_O· + O_2_
    CHCl_2_O· + O_2_ → COCl_2_ + HO_2_
    CH_2_ClO· + HO_2_ → COCl_2_ + H_2_O_2_
3. Chloroform (R=CCl_3_)
  (1) CCl_3_O· → COCl_2_ + Cl
  (2) CCl_3_O· → COCl_2_ + ClO
    2CCl_3_O_2_· → 2COCl_2_ + Cl_2_ + O_2_

In the case of methylene chloride, H_2_O_2_ may be a degradation product. However, H_2_O_2_ acts as a reservoir molecule for HO_x_ in the troposphere, showing its concentrations in the range of 2–6 ppb [36]. In this regard, this source will be not significant compared with other atmospheric sources of H_2_O_2_ in the CO oxidation cycle. On the other hand, molecular chlorine (Cl_2_) can be formed from the photochemical degradation of chloroform. This formation reaction would be negligible due to bimolecular decomposition, in comparison with other loss processes of CCl_3_O_2_· [41]. 

### 3.3. Hazards of Degradation Products

According to the discussion above, the main degradation products of common chloromethanes in the atmosphere include HCl, CO, CO_2_, Cl_2_, HCOCl (formyl chloride), COCl_2_, and H_2_O_2_. It should be noted that formyl chloride will thermally decompose to HCl and CO at room temperature. Moreover, Cl_2_ and COCl_2_ will be easily absorbed into humid aerosols, further forming HCl [42]. The limits of human exposure to these degradation products from chloromethanes established or recommended by the governmental or non-profit organizations are listed in Table 6. Among them, carbonyl chloride (COCl_2_) is a powerful irritating gas. This photochemical by-product is easily hydrolyzed or thermally decomposed to form hydrochloric acid [43], which is highly toxic to humans by inhalation due to the release of chloride ions [26].

## 4. Conclusions

Among halogenated hydrocarbons, chloromethanes play a vital role from both industrial and commercial standpoints, not only as extensive solvents, but also as important chemical intermediates. Although carbon tetrachloride (CCl_4_) has been included in the basket of the Montreal Protocol for eliminating its production and use, the other three chloromethanes (i.e., methyl chloride, CH_3_Cl; methylene chloride, CH_2_Cl_2_; and chloroform, CHCl_3_) are volatile organic compounds (VOCs), thus posing some potential hazards to the atmospheric environment due to their toxicological and environmental properties. In this review, the environmental properties of chloromethanes and their atmospheric fates indicate that most of them will be present in the atmosphere under normal conditions. When released into the atmosphere, these compounds will react with highly-oxidative species (e.g., NO) and free radicals (e.g., hydroxyl radicals) in the troposphere and are unlikely to diffuse into the stratosphere. As a consequence, the end degradation products in the troposphere will contain toxic compounds, including HCl, CO, Cl_2_, COCl_2_, and H_2_O_2_. On the other hand, its impacts on the global climate change and stratospheric ozone depletion may be more significant in the future because of their various industrial/commercial uses. In view of their extensive uses in the metal cleaning and vapor degreasing processes, several clean technologies, such as aqueous cleaning, emulsion cleaning, supercritical fluid (SCF), and media blasting, are being offered to industry as the alternatives to methylene chloride and chloroform.

## Figures and Tables

**Table 1:** Identification and physical properties of chloromethanes.

Property	Units	CH_3_Cl	CH_2_Cl_2_	CHCl_3_	CCl_4_
IUPAC name	—	Chloromethane	Dichloromethane	Trichloromethane	Tetrachloromethane
Common name	—	Methyl chloride	Methylene chloride	Chloroform	Carbon tetrachloride
CAS number	—	74-87-3	75-09-2	67-66-3	56-23-5
Molecular weight	g/mol	50.5	84.9	119.4	153.8
Relative vapor density (air = 1)	--	1.75	2.93	4.12	5.32
Boiling point at 1 atm	°C	−23.7	39.8	61.3	76.6
Freezing point at 1 atm	°C	−97.7	−96.7	−63.5	−22.8
Critical temperature	°C	143.1	237.0	263.4	283.3
Critical pressure	MPa	6.679	6.171	5.500	4.557
Critical density	kg/m^3^	353	472	500	558
Dipole	Debye	1.9	1.8	1.1	0.0
Density 20 °C	g/cm^3^	0.997 (−24 °C)	1.322	1.490	1.595
Viscosity (20 °C)	mPa.s	0.106 (gas)	0.430	0.563	0.965
Vapor pressure (20 °C)	kPa	506.1	46.5	21.3	11.9
Refractive index (20 °C)	—	1.3712 (−23.7 °C)	1.4244	1.4467	1.4631
Latent heat of vaporization at b.p.	kJ/kg	424.1	330.0	247.0	194.7
Log Pow (20 °C)	g/mol	0.91	1.25	1.97	2.83
Water solubility (20 °C)	mg/L	6310	13,000	7950	805
Henry’s Law constant (25 °C)	atm-m^3^/mol	0.024	0.00268	0.00435	0.0302
Flammability limits	Vol %	8.1–17.4	14-25	--	--

**Table 2:** Hazards of chloromethanes to human health.

Compound	NIOSH ^a^		TLV Basis- Critical Effect ^b^
	Exposure Routes	Target Organs	
CH_3_Cl	Inhalation, skin and/or eye contact (liquid)	Central nervous system (CNS), liver, kidneys, reproductive system	CNS impair; liver, kidney, and testicular damage; teratogenic effects
CH_2_Cl_2_	Inhalation, skin absorption, ingestion, skin and/or eye contact	Eyes, skin, cardiovascular system, CNS	COHb-emia; CNS impair
CHCl_3_	Inhalation, skin absorption, ingestion, skin and/or eye contact	Liver, kidneys, heart, eyes, skin, CNS	Liver and embryo/fetal damage; CNS impair
CCl_4_	Inhalation, skin absorption, ingestion, skin and/or eye contact	CNS, eyes, lung, liver, kidneys, skin	Liver damage

^a^ The data are from [26]; ^b^ The data are from [27].

**Table 3:** Occupational exposure limits of chloromethanes.

Compound	Exposure Limits				
	TLV ^a^	PEL ^b^	IDLH ^c^	MAK ^d^	PCS ^e^
CH_3_Cl	50 ppm	100 ppm	2000 ppm	50 ppm	50 ppm
CH_2_Cl_2_	50 ppm	25 ppm	2300 ppm	50 ppm	50 ppm
CHCl_3_	10 ppm	50 ppm (Ceiling)	500 ppm	0.5 ppm	10 ppm (Ceiling)
CCl_4_	0.1 ppm	10 ppm	200 ppm	0.5 ppm	2 ppm

^a^ Threshold limit value (ACGIH, Cincinnati, OH, USA); ^b^ Permissible exposure limit (OSHA, Washington, DC, USA); ^c^ Immediately dangerous to life or health (NIOSH, Washington, DC, USA); ^d^ Maximum concentrations at the workplace (DFG, Bonn, Germany); ^e^ Permissible exposure limit (OSHA, Taipei, Taiwan).

**Table 4:** Carcinogenicity classification of chloromethanes.

Compound	Carcinogenicity Classification/Category				
	IARC	UN EPA	US NTP	ACGIH	DFG
CH_3_Cl	3	-- ^a^	-- ^a^	--	3B
CH_2_Cl_2_	2A	likely to be carcinogenic	Reasonably anticipated to be human carcinogens	A3	5
CHCl_3_	2B	likely to be carcinogenic	Reasonably anticipated to be human carcinogens	A3	4
CCl_4_	2B	likely to be carcinogenic	Reasonably anticipated to be human carcinogens	A2	4

^a^ Not available.

**Table 5:** Environmental properties of chloromethanes.

Compound	Atmos. Lifetime ^a^ (yr)	Radiative Efficiency ^b^ (W m^−2^ ppb^−1^)	GWP ^c^	ODP ^d^	POCP ^e^	Surface Mixing Ratio ^f^ (ppt)
CH_3_Cl	1.0	0.01	12	0.02	1	530–560
CH_2_Cl_2_	0.4	0.03	9	≈0.0	3	20–60
CHCl_3_	0.4	0.08	16	≈0.0	≈0	10–20
CCl_4_	33.0	0.17	1730	1.1	0	80–90

^a^ Source [33]; ^b^ Source [33,34]; ^c^ Global warming potential with a 100-year time horizon (relative to GWP of CO_2_ = 1.0); source [33]; ^d^ Ozone depletion potential (relative to the ODP of CFC-11 = 1.0); source [13,32]; ^e^ Photochemical ozone creation potential (relative to POCP of ethene = 100); source [31]; ^f^ Source [13,30,32].

**Table 6:** Hazards of degradation products of chloromethanes to human health.

Degradation Products	UN NIOSH ^a^		TLV Basis- Critical Effect ^b^
	Exposure Routes	Target Organs	(TLV)
Cl_2_	Inhalation, skin and/or eye contact	Eyes, skin, respiratory system	Upper respiratory tract (URT) and eye irritation (0.5 ppm-TWA)
HCl	Inhalation, skin and/or eye contact, ingestion (solution)	Eyes, skin, respiratory system	URT irritation (2 ppm-ceiling)
COCl_2_	Inhalation, skin and/or eye contact (liquid)	Eyes, skin, respiratory system	URT irritation; pulmonary edema (0.1 ppm-TWA)
CO	Inhalation, skin and/or eye contact (liquid)	Cardiovascular system, lungs, blood, central nervous system	COHb-emia (25 ppm)
CO_2_	Inhalation, skin and/or eye contact (liquid/solid)	Respiratory system, cardiovascular system	Asphyxia (5000 ppm)
H_2_O_2_	Inhalation, skin and/or eye contact	Eyes, skin, respiratory system	Eye, URT, and skin irritation (1 ppm)

^a^ The data are from [26]; ^b^ The data are from [27].
